# To Lyse or Not to Lyse: Transient-Mediated Stochastic Fate Determination in Cells Infected by Bacteriophages

**DOI:** 10.1371/journal.pcbi.1002006

**Published:** 2011-03-10

**Authors:** Richard I. Joh, Joshua S. Weitz

**Affiliations:** 1 School of Physics, Georgia Institute of Technology, Atlanta, Georgia, United States of America; 2 School of Biology, Georgia Institute of Technology, Atlanta, Georgia, United States of America; University of Texas at Austin, United States of America

## Abstract

Cell fate determination is usually described as the result of the stochastic dynamics of gene regulatory networks (GRNs) reaching one of multiple steady-states each of which corresponds to a specific decision. However, the fate of a cell is determined in finite time suggesting the importance of transient dynamics in cellular decision making. Here we consider cellular decision making as resulting from first passage processes of regulatory proteins and examine the effect of transient dynamics within the initial lysis-lysogeny switch of phage *λ*. Importantly, the fate of an infected cell depends, in part, on the number of coinfecting phages. Using a quantitative model of the phage *λ* GRN, we find that changes in the likelihood of lysis and lysogeny can be driven by changes in phage co-infection number regardless of whether or not there exists steady-state bistability within the GRN. Furthermore, two GRNs which yield qualitatively distinct steady state behaviors as a function of phage infection number can show similar transient responses, sufficient for alternative cell fate determination. We compare our model results to a recent experimental study of cell fate determination in single cell assays of multiply infected bacteria. Whereas the experimental study proposed a “quasi-independent” hypothesis for cell fate determination consistent with an observed data collapse, we demonstrate that observed cell fate results are compatible with an alternative form of data collapse consistent with a partial gene dosage compensation mechanism. We show that including partial gene dosage compensation at the mRNA level in our stochastic model of fate determination leads to the same data collapse observed in the single cell study. Our findings elucidate the importance of transient gene regulatory dynamics in fate determination, and present a novel alternative hypothesis to explain single-cell level heterogeneity within the phage *λ* lysis-lysogeny decision switch.

## Author Summary

Multicellular organisms, single-celled organisms, and even viruses can exhibit alternative responses to various internal and environmental conditions. At the cellular level, alternative fate determination is usually described as the result of the inherent bistability of gene regulatory networks (GRNs). However, the fate of a cell is determined in finite time suggesting the importance of transient dynamics to cellular decision making. Here, we present a quantitative gene regulatory model of how bacteriophages determine the fate of an infected bacterium. We find that increasing the number of infecting phages increases the chance of quiescent (i.e., lysogeny) vs. productive (i.e. lysis) viral growth, in agreement with prior studies. However, unlike previous theoretical studies, the bias in cell fate is a result of the transient divergence of stochastic gene expression dynamics. We compare and contrast our theoretical model with recent observations of cell fate measured at the single-cell level within multiply-infected cells. Predicted heterogeneity in cell fate is shown to agree with data when including a previously unidentified gene dosage compensation mechanism, which represents an alternative hypothesis to how multiple phages interact in influencing cell fate. Together, our results suggest the importance of quantitative details of transient gene regulation in driving stochastic fate determination.

## Introduction

Biochemical pathways and feedbacks in gene regulatory networks (GRNs) shape when and how much genes are expressed. Differential gene expression can lead to qualitative changes in cellular phenotypes, whether via alternative cell fate determination in unicellular organisms (e.g., competence [1], sporulation [2], persistence [3], and infected cell fate [4]) or via cell differentiation in multi-cellular organisms (e.g., lineage determination [5]). The steps leading to qualitative changes in phenotype are not strictly deterministic. Gene regulation is an inherently noisy process involving transcription control, translation, diffusion and chemical modifications of transcription factors, all of which may be characterized by stochastic fluctuations due to low copy numbers of regulatory molecules [6]–[8]. As a result, genetically identical cells can have marked differences in the state of regulatory molecules even when faced with identical environmental conditions [9]–[11]. Explanations for alternative cell fate determination generally presume the existence of multiple stationary states within the GRN [12], [13]. Determination of cell fate is therefore usually described as the result of the interplay between noise and deterministic dynamics of GRNs which determines the relative frequency of each decision [12], [14].

A potential problem with this explanation is that cellular decision making occurs within finite time. From a theoretical point of view, differences in asymptotic dynamics are not necessary for regulatory dynamics to reach markedly different transient states. The hypothesis that transient dynamics can drive cell fate determination has been suggested in the context of HIV-1 latency where a bistable response is observed despite the purported monostability of the GRN [15]. Here, we take a generalized approach to a similar problem by considering cell fate determination as the result of stochastic transient dynamics of a GRN. Our starting point is the fact that extrinsic variation can drive substantial differences in the transient state of regulatory molecules [16]. That is to say, ensembles of cells with the same initial state of regulatory molecules which are exposed to two different conditions can follow distinct transient trajectories on average. In such a case, gene expression will be characterized by an early period in which transient trajectories are unresolvable with respect to the stochastic noise and a middle period in which they are markedly different. However, we claim that such transient differentiation in regulatory state need not be accompanied by marked differences in asymptotic, i.e., steady-state, behavior. Instead, we hypothesize that alternative cell fate decisions can be mediated by first passage processes of regulatory molecules [17], [18].

We examine the effect of first passage processes in stochastic GRNs within the initial decision switch between lysis and lysogeny by phage 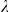. Bacteriophage 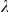 is perhaps the simplest example of an organism with alternative developmental modes, which are quiescent (lysogenic) and productive (lytic) growth upon infecting *E. coli* cells [4], [19]–[22]. Here we focus on how phage-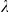-infected cells are lysed or become lysogens as a function of the number of coinfecting phages (also known as the cellular multiplicity of infection denoted as 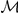). Experimental infection assays have revealed that *E. coli* cells that are multiply infected tend to become lysogens whereas singly infected cells tend to be lysed [23], [24]. The decision to lyse a cell or enter lysogeny is stochastic [25], [26], and the fraction of lysogeny is a probabilistic function of the number of coinfecting phages and cell volume [27]–[29]. Cells that become lysogenic may later spontaneously induce leading to virion production and cell lysis. The stability of the lysogenic state has also been evaluated in light of first exit problems [30], many of whose concepts we adapt in the current model of the initial decision switch.

A significant advantage in modeling phage 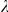 is that the core pathways of lysis-lysogeny have been studied extensively. Subsequent to infection, the repressors (CIs) bind cooperatively to adjacent operator sites [31], and cooperative binding can induce DNA loops which enhance the stability of the lysogenic state [32]–[34]. Early quantitative studies of the initial lysis-lysogeny decision utilized statistical thermodynamic models which described the dynamics of gene regulation by cooperative binding of CI [35], [36]. Arkin *et. al.* developed a fully stochastic model based on transcription, translation and protein interactions [25]. Whether cells were fated to lysis or lysogeny was ascribed to intrinsic stochasticity, whose complexity rendered it intractable for mathematical analysis. More recently, theoretical work has suggested that alternative decisions of lysis and lysogeny may be due to inherent bistability of the phage 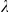 GRN with respect to changes in copy number concentration (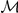 divided by host cell volume) [28]. However, this model presumes that differences in asymptotic dynamics lead to changes in cell fate, without considering stochastic effects in transient dynamics.

In this study, we demonstrate that biased alternative cell fate decisions are possible due to transient divergences within gene regulatory dynamics. As evidence, we develop and analyze a quantitative model of a GRN of phage 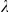 based on empirical analyses of viral infection. Although the structure of the phage 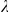 GRN is relatively well established, the quantitative values of most kinetic parameters involved in viral gene regulation remain either unknown or poorly constrained. We examine two sets of kinetic parameters close to consensus empirical estimates which we refer to as transiently divergent and asymptotically divergent, respectively. We show that the dynamics of the GRN with these parameter sets are similar shortly after phage infection but the asymptotic dynamics are qualitatively distinct as a function of viral genome concentration. Next, we compare the fraction of lysogeny as a function of viral genome concentration in the two parameter sets. Cell fate is determined via first passage processes of two regulatory proteins, Q and CI, corresponding to lysis and lysogeny, respectively (see Models). We find that equivalent responses of cell fate to changes in viral genome concentration can be obtained with either parameter set, suggesting caution must be applied in interpreting alternative cell fate determination as a hallmark of bistability. In the process, we also discuss how thresholds of first passage processes can change the fraction of lysogeny and the time scale of decisions. Finally, we compare model results with experimental data on cell fate outcomes from single cell assays [29]. We propose an alternative data collapse of the observed cell fate outcomes, consistent with a previously unidentified gene dosage compensation mechanism. We show that including gene dosage compensation at the mRNA level in our stochastic model of transient fate determination also leads to the form of data collapse observed in the single cell study. We conclude by discussing means to reconcile multiple competing hypotheses for observed heterogeneity in the phage 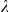 GRN.

## Results

### Deterministic dynamics of qualitatively identical phage 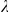 decision switches can be asymptotically or transiently divergent

**Figure 1:**
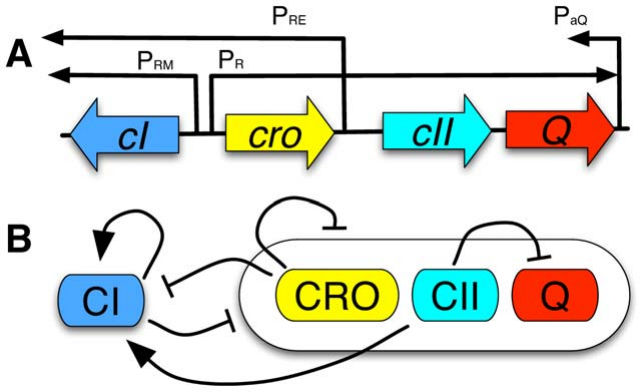
Core genetic components of lysis-lysogeny decision switch in phage 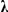. (A) Schematic diagram of genes and promoters. CI and CRO dimers are the transcription factors for 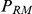 and 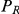 while 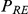 and 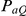 is controlled by CII tetramers. Black arrows represent open reading frames of promoters when activated (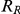, 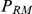 and 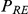) and antisense transcript *aQ*. (B) Interactions among gene products. Regular and blunt arrows represent positive and negative feedbacks, respectively. CI dimers are self-activators while repressing the other genes, and CRO dimers repress all the genes in the system. CII tetramers activate *cI* transcription, and suppress Q expression by transcribing antisense mRNAs.

**Figure 2:**
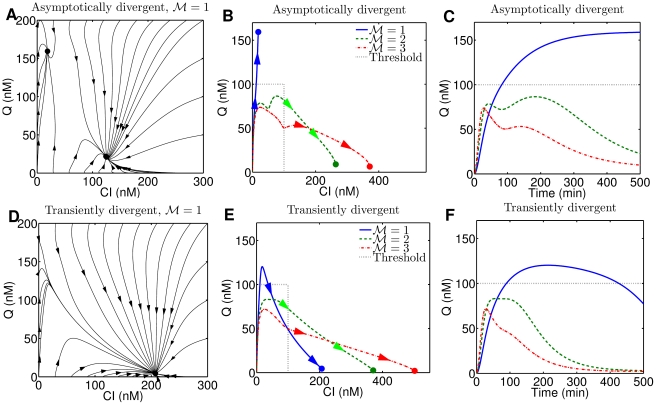
Dynamics of regulatory proteins, CI and Q, when the GRN is asymptotically divergent and transiently divergent. (A) Phase diagram of CI-Q dynamics when asymptotically divergent for 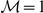. Note that the system is bistable. (B) Phase diagram of CI-Q dynamics starting from no viral proteins when asymptotically divergent. Thresholds of CI and Q (both at 100 nM) represent the concentrations above which decisions are lysogenic and lytic, respectively. Trajectories cross the threshold only once. (C) Asymptotically divergent dynamics of Q concentration as a function of time. (D) Phase diagram of transiently divergent system with 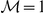. Note that the system is not bistable. (E) Phase diagram of CI-Q dynamics of the transiently divergent phage 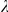 GRN. At 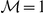 the deterministic trajectory crosses the threshold three times, and decisions change from lysis to lysogeny as a function of time. (F) Transiently divergent Q dynamics.

We first analyze a deterministic model of a GRN of phage 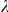 (see Fig. 1, and Models Eq. (3)). Prior to phage 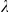 infection, there are no viral proteins and mRNAs in the host cell. A cell can be infected by 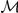 phages, which we vary one to five for a fixed cell volume. Cell fate, either lysis or lysogeny, is determined based on the first passages of a pair of fate-determining regulatory molecules, CI and Q (see Fig. 2 (B,E)). We model lysogeny as occurring when CI exceeds a concentration threshold and lysis as occurring when Q exceeds a concentration threshold. We set the value of these thresholds at 100 nM each, and explore the impact of varying these thresholds levels. Values of kinetic parameters necessary for modeling the lysis-lysogeny decision switch are known to within a few percent error in some cases, unknown in other cases, or have estimates with significant uncertainty (see Table 1). We chose two sets of parameters which are close to the consensus estimates, but that show markedly distinct asymptotic behaviors especially when 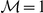. GRNs with these two sets are asymptotically and transiently divergent, respectively (Fig. 2). We define a phage 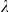 GRN with a set of kinetic parameters to be asymptotically divergent if each deterministic trajectory for 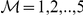 crosses the CI and Q thresholds only once. Otherwise, a GRN is referred to as transiently divergent.

**Table 1:** Parameters for transiently divergent and asymptotically divergent GRNs.

Parameter	Reference value	Reference	Asymptotically divergent	Transiently divergent
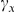	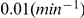	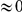 [66], 0.042 [25]	0.014	0.013
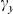	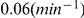	0.016 [67]	0.033	0.056
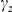	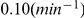	0.16 w/o CIII [68]	0.13	0.22
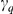	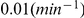		0.0095	0.016
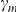	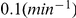	0.12 [69]	0.1	0.1
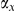	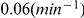	0.06 [36]	0.055	0.055
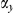	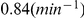	0.84 [36], 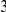 [66]	0.82	0.70
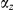	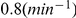	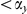	0.50	0.83
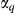	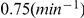	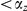	0.78	0.63
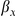	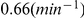	0.66 [36], 3.42 [70]	0.79	0.88
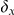	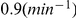	0.9 [25]	1.24	0.93
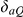	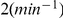		3.1	3.8
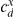	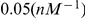	0.05 [71], 0.18 [72]	0.060	0.079
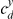	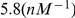	5.8 [73], 307 [74]	4.6	6.7
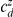	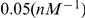		0.065	0.020
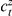	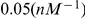		0.093	0.068
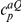	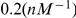		0.14	0.10
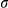	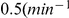 )		0.38	0.47
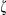	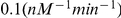	0.02 [75]	0.15	0.068
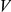	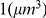	0.5 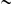 2.0		

The transient dynamics for the phage 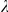 GRN given either parameter set (either asymptotically or transiently divergent) are similar during the time scale of lysis-lysogeny decision (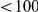
min). The asymptotically divergent phage 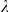 GRN exhibits lysis for 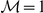 and lysogeny when 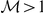. Note that the ratio between CI and Q changes dramatically as a function of 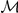 from having far more Q to having far more CI at steady state. Only when 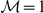 are there two possible steady states, but the initial condition leads to lysis (Fig. 2 (A)). In contrast, the transiently divergent GRN is monostable for all values of 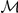 that we considered. Further, the steady-state CI and Q concentrations have far greater levels of CI than Q, suggesting that an asymptotic analysis would suggest that the transiently divergent GRN would always lead to lysogeny. However, note that when 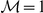, Q increases rapidly, exceeds the threshold for lysis, and only later does it drop down and approaches a case where Q is low and CI is high (Fig. 2 (D–F)). Thus, there is an inconsistency between expectations for cell fate determination as viewed in finite time vs. that viewed asymptotically.

### Alternative cell fates as determined by transient viral gene regulation

**Figure 3:**
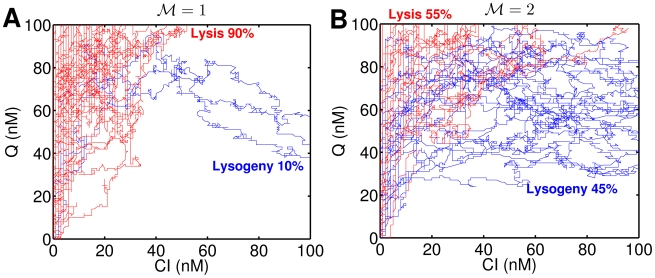
Stochastic realization of C and Q dynamics for (A) 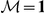 and (B) 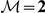. Trajectories are sampled for every 1/4 minute. The system is transiently divergent, and thresholds are set at 100 nM for both CI and Q. Each curve represents a single realization, and 50 realizations are shown here. Red trajectories indicate that decisions are lytic whereas blue ones represent lysogeny.

The initial lysis-lysogeny decision of phage 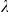 is sensitive to the external conditions of 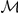 and cell size. Empirical analyses have shown this decision to be highly stochastic with the fraction of lysogeny between 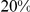 and 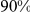 for physiologically relevant 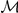 and cell size [29]. To model the stochastic nature of this decision, we assume that first passage processes of CI and Q determine whether lysis or lysogeny occurs in an infected cell. Lysogeny occurs if CI reaches its critical concentration before Q does. Lysis occurs if the opposite holds true. We follow the approach of Arkin *et. al.* [25] and run fully stochastic simulations of the phage 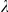 GRN while setting both lytic and lysogenic thresholds at 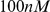 (see Models). We assume that reaching a decision of lysis or lysogeny brings a topological change to the GRN. Thus, we stop the dynamics at the time of a decision since our phage 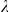 model cannot describe the post-decision regulatory dynamics. Fig. 3 depicts a subsample of trajectories in the phase space of CI-Q labeled according to which decision is reached via a first passage process. Note that there is a delay for CI to be expressed since sufficiently abundant CII is required for initial CI expression. In contrast, Q can be produced immediately after phage infection. When the host is singly infected, lysis is the dominant decision, and CI does not build up until a significant amount of Q is produced (Fig. 3 (A)). At higher 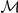 (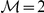 for Fig. 3 (B)), CII and Q are produced at a higher rate. Depending on the CII expression level Q can be repressed while CI becomes active which leads to lysogeny. In comparison to the deterministic dynamics described in the previous section, there is significant variability in the lysis-lysogeny bias of the GRN, though the bias itself is affected by changes in 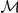 and cell volume (as described in the next section).

### Probability of lysogeny is an increasing function of phage genome concentration

**Figure 4:**
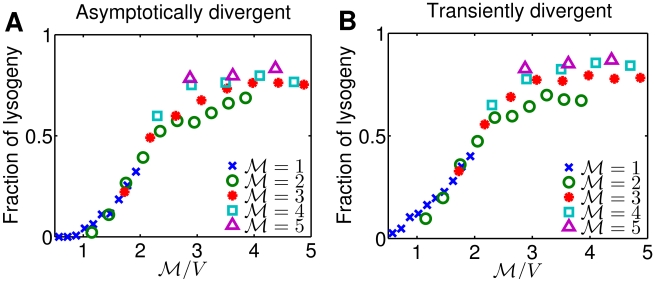
Response of phage 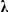 to various phage genome concentrations when (A) asymptotically divergent and (B) transiently divergent. 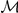
 and 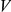 represent the number of coinfecting phages and the host cell volume, respectively, so 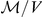 is the phage genome concentration. Each point is the result from 5,000 simulations.

We vary the volume of host cells (denoted as 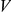) as well as 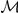 in order to investigate how cell fate responds to changes in the concentration of viral genomes (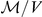). For consistency with experimental studies and to model physiologically reasonable values, we vary 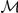 from one to five, and vary 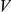 from 0.5 to 2 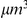. Fig. 4 shows the fraction of lysogeny as a function of phage genome concentration. Regardless of bistability in the phage 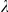 GRN, we find that first passage mediated decision making can lead to systematic biases in alternative cell fate determination. Phages preferentially enter lysogeny when multiple phages infect the same hosts while singly infected hosts tend to be fated for lysis. The relative frequencies of lysis or lysogeny can be collapsed as a function of an extrinsic parameter 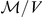. Our results match the general trend of recent experimental observations which demonstrated that the fraction of lysogeny goes up as phage genome number increases or cell volume decreases [27], [29]. Importantly, the functional responses to phage genome concentration are nearly indistinguishable even for two parameter sets which have qualitatively different asymptotic dynamics (Fig. 4 and Fig. 2 (B,E)). The biased decision response as a function of phage genome concentration is due to the similarity of transient dynamics, irrespective of asymptotic dynamics that could have been followed. Hence, the finding that infected cell fate can change from predominantly lytic (at 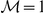) to predominantly lysogenic (at 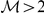) is not necessarily a hallmark of an underlying bistable viral GRN nor of a bifurcation in the underlying dynamics as a function of 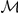 or 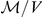. Despite the agreement with prior empirical studies, note that our model does not predict systematic decreases in the lysogen fraction given a fixed value of 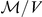 and increasing values of 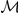, as observed in a recent single-cell experimental study [29]. In the next section, we revisit the experimental data from Zeng *et. al.* [29] and in so doing, provide an alternative data collapse and a corresponding mechanism that is consistent with a modified version of the current stochastic model.

### Mechanism of partial gene dosage compensation accounts for observed heterogeneity in lysis-lysogeny decisions

**Figure 5:**
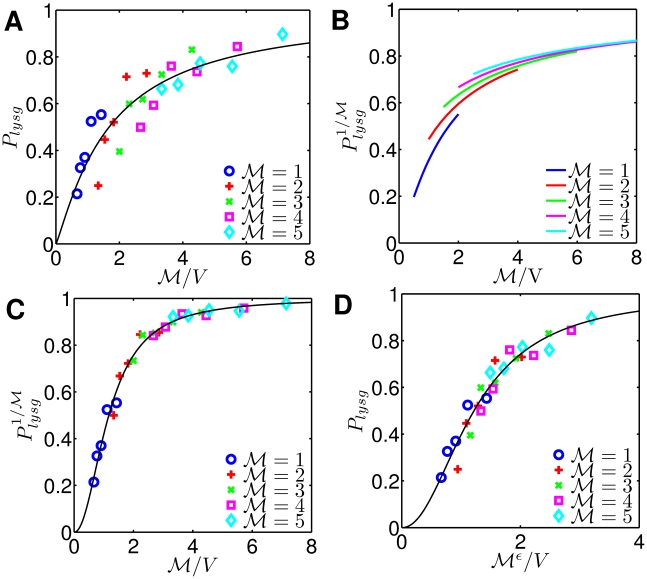
Alternative mechanisms underlying heterogeneity of lysis-lysogeny decisions. (A) Fraction of lysogeny plotted from single cell assays[29]. (B) Rescaled probability of 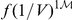. Each phage within a host is completely independent from other phages, and decision of lysogeny becomes a function of host volume. Note that rescaled curves do not collapse into a single curve. (C) Rescaled probability of 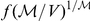 proposed by Zeng *et. al.* [29] representing the probability of lysogeny for each individual infecting phage. Each phage independently “chooses” lysis or lysogeny. However, since the fraction of lysogeny for a single phage is a function of 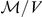, phages sense the presence of other phages. Note that data from different 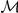-s collapse into a single curve. (D) Probability of lysogeny plotted against rescaled 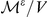 when 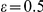, corresponding to a mechanism in which gene expression from multiple copies is partially compensated. Due to partial dosage compensation, the transcription rate is not linearly proportional to 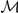, and the effective copy number is given as 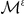 where 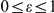. Note that the data from different 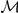-s collapse into a single curve. Black lines represent nonlinear curve fits into Hill functions.

Zeng *et. al.* [29] measured the fate of multiply infected cells in which the number of phages and cell volume could be measured on a per-cell basis. The experimental protocol induces viral injection with an abrupt change in temperature and hence, infections are treated as simultaneous. The experimental data demonstrate that the fraction of lysogeny increases with viral concentration, 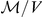 (Fig. 5 (A)). This trend agrees with prior experimental works showing that increases in co-infection number increases the likelihood of lysogeny [23], [24] and that increases in cell volume increases the likelihood of lysogeny [27]. However, there is significant amount of heterogeneity in the observed cell fate data other than strict dependence on 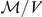 as suggested by theory [28].

In particular, Zeng *et. al.* [29] observed that the fraction of lysogens decreases with increasing 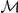 for a given ratio of 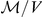. Zeng *et. al.* [29] suggested that the remaining heterogeneity in cell fate not explained by a strict dependence on 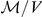 is due to a voting mechanism that takes place at the single-cell level. In this view, a unanimous decision of phages is required by phages for lysogeny [29] (presumably because a single phage that is fated to lysis would over-ride a decision by other phages for lysogeny). If each coinfecting phage is totally independent from each other, then one would expect the probability of lysogeny to be: 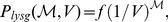
(1)


where 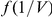 is the probability that a cell of volume 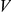 infected by a single phage would become a lysogen. Fig. 5 (B) shows the fraction of lysogeny scaled with 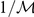 power based on the empirical observations for the singly infected case. The re-scaled data for the five values of 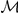 should agree with 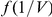 in an independent phage voting model. However, this rescaling does not form a single line. This suggests that there might be some inter-dependence between phages.

Indeed, the voting model proposed by Zeng *et. al.* [29] is actually a “quasi-independent” voting model. In this view, a unanimous decision of phages is required by phages for lysogeny [29]. However, the probability that any given phage decides for lysogeny becomes a function of the viral genome concentration, 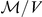. Thus the fraction of lysogeny becomes 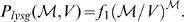
(2)


where 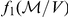 is the probability that a single phage reaches a lysogenic decision state given that it is in a cell of volume 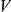 with a total of 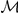 phages. The re-scaled probability of entering lysogeny at the whole cell level, 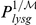, is shown in Fig. 5 (C). Notably, the re-scaled experimental data collapses on a single line, presumably 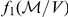. Thus, this mechanism captures the characteristics of experimental data phenomenologically. However, the mechanism involves both independence and inter-dependence among phage genomes that remains un-identified at the subcellular level.

Here, we revisit the cell fate data of Zeng *et. al.* [29] and propose a mechanism of partial gene dosage compensation as an alternative explanation for the scaling collapse they observe. In this context, partial gene dose compensation means that a cell with multiple copies of a viral genome has smaller per-copy viral gene expression than a cell with a single viral genome. Indirect support already exists for this hypothesis. For example, Zeng *et. al.* [29] showed that the fraction of cells with halted growth increases with the number of co-infections, suggesting that viral genomes have adverse effects on cellular metabolism in addition to or instead of lysis. Earlier studies showed that phage 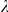 infections repress host synthesis activity at the level of transcription [37] and translation [38]. The degree of repression depends on the number of coinfections, and more coinfections lead to greater repression. Broadly speaking, the mechanism (or mechanisms) underlying gene dosage compensation remains an open question. However, it has been widely noted that copy numbers of genes and chromosomes can differ among cells and individuals, but the resulting gene expression need not be a linear function of gene copy number [39]–[41].

Here, we assume that partial gene dosage compensation occurs at the level of transcription. Specifically, we assume that the total transcription rate of a gene is proportional to 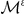 where 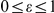 (see Models and Eq. (3)). 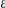 is the quantitative measure of partial gene dosage compensation and RNA synthesis repression by phage genomes. When 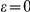, increases in viral genome have no effect on transcriptional rates, whereas when 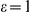, transcriptional rates increase linearly with 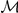 (as in the original model described previously in this paper). Hence, if a partial gene dosage mechanism is at work, then the lysogeny data should collapse when plotted against 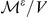. Fig. 5 (D) shows the fraction of lysogeny against 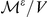 which incorporates the effect of partial gene dosage compensation. Note that the data collapses into a single line, similar to the quasi-independent decision mechanism. The estimate of 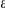 from experimental data is about 0.5, suggesting that the overall viral transcriptional activity in the host cells on a per-viral genome basis scales with 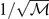.

**Figure 6:**
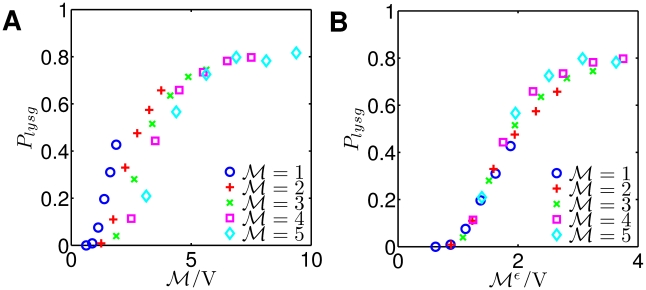
Effect of gene dosage compensation from stochastic simulations. (A) Fraction of lysogeny from stochastic simulations. Simulations with partial dosage compensation exhibit the nested pattern of 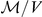 dependence as seen in the experimental data (see Fig. 5). (B) Simulation results on the fraction of lysogeny from Fig. 6 (A) plotted with rescaled 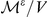 when 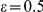. The outcome of stochastic simulations with partial dosage compensation is consistent with experimental data (see Fig. 5 (A,D)). In this case, the GRN is asymptotically driven with CI and Q threshold at 100 nM and 120 nM, respectively, all other parameters are set according to Table 1 - transiently divergent. Each point is the result from 3,000 simulations.

Hence, two distinct mechanisms: (i) quasi-independent decision making; and (ii) partial gene dosage compensation, can explain heterogeneous decision making from single cell assay based experiments using data collapse. Note that we cannot evaluate the quasi-independent mechanism using our model because doing so would require incorporating genome-specific changes (such as anti-termination events) or compartmentalizing the cell with respect to transcription and translation events (requiring even more unknown parameters than the current model). However, it is possible to explicitly incorporate partial gene dosage compensation in stochastic simulations (see Models). In brief, we modified transcriptional rates so that transcription increased with 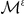 instead of 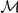 and ran stochastic simulations with all other parameters as before. Fig. 6 shows the fraction of lysogeny resulting from the stochastic fate determination model incorporating partial gene dosage compensation against 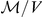 and rescaled 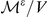. Stochastic simulations with partial dosage compensation exhibit the heterogeneous, yet strong dependence of lysogeny on 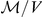. Moreover, the cell fate results of stochastic simulations collapse into a single line when 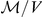 is rescaled as 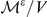. Given the new scaling collapse, cells with the same 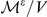 have a lower chance of lysogeny given increasing values of 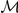, consistent with the pattern observed in the experimental study (Fig. 5 (D)). Hence, we propose that partial gene dosage compensation should be considered as an alternative mechanism to explain the heterogeneous cell fate of bacteria infected by bacteriophage 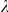.

## Discussion

In this paper, we have proposed and analyzed a transient mechanism of cell fate determination in terms of first passage processes of regulatory proteins. We applied this mechanism to the study of the initial lysis-lysogeny decision in bacterial cells infected by phage 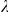. We found that stochastic simulation of parametrized viral GRNs lead to changes in the the frequency of alternative fates for infected cells, either lysis or lysogeny, as a function of the genome concentration of infecting viruses. The biased response in cell fate outcome occurs despite intrinsic noise in the system and does not require the bistability of the underlying GRN. Hence, alternative and seemingly adaptive cell fate decisions may be due to transient divergence in stochastic trajectories of regulatory molecules and not necessarily due to underlying bistability. Finally, we showed that a partial gene dosage compensation is a candidate mechanism underlying noise in lysis-lysogeny decisions, as supported by both our quantitative model and experimental data.

Our central result is in contrast to the conventional perspective that multistability is required for alternative decisions [13], [14]. Multistability often requires cooperative binding as a necessary condition for the emergence of the two or more stable steady states in the GRN [42], [43]. A recent study showed that a switch system can arise in the absence of cooperative bindings [44]. Our study suggests that cooperative binding may occur and affect transient dynamics but not necessarily lead to bistability in asymptotic dynamics. Together these results suggest that GRNs which do not have bistability or cooperative bindings might be able to lead to alternative cell fate determinations. Thus, it might be possible for a GRN to evolve (by natural selection) or to be designed (via synthetic means) to perform a complex task of alternative decision making in response to external stimuli without multistability. Note that such a transiently excitable GRN which differentiates transient and asymptotic phenotypes was experimentally demonstrated in *Bacillus subtilis* [45]. Generally, there exist examples of GRNs which are responsive to environmental signals and robust to changes of kinetic parameters [46] while other are sensitive to kinetic parameters. Sensitivity of transient dynamics to a GRN's kinetic parameters and thresholds might be a target of selection over evolutionary time scales. In this context, we examined how modifying thresholds for decisions can lead to systematic changes in lysis vs. lysogeny as well as decision times (see Fig. S2). The general result from the present analysis is that alternative determination requires separation of thresholds, which comes at the expense of slower decisions. Hence, transiently driven cellular decisions have the potential to be highly evolvable.

As we have detailed, stochastic simulations of the phage GRN proposed here can reproduce a number of characteristics for how the fraction of lysogeny changes with 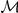 and cell volume. Importantly, we find that lysogeny increases with increasing 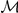 [23], [24] and decreasing cell volume [27], and remains between approximately 20%–90% for physiologically reasonable values [29]. The bias in cell fate outcome in favor of lysogeny with increasing 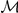 may be adaptively significant. On average, high 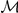 implies that phages infect hosts frequently on the time-scale of decision-making and further, that phages are more abundant than their bacterial hosts. Lysis will further increase the phage-host ratio, and a previous study has speculated that phages seem to avoid depletion of hosts by entering lysogeny predominantly at high 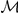 [47]. However, if lysogeny is adaptively favorable at high 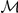, why is it that a small fraction of phages still enter the lytic pathway? The answer could be due to constraints in the resolvability of the GRN due to the strength of intrinsic stochasticity in the GRN [29]. Or the stochasticity itself may be adaptive. Phages may have evolved to respond to changes in intracellular phage genome concentration in order to minimize the chance of extinction [48] by maintaining phage and lysogen population as a bet-hedging strategy [49]. Any such speculations require careful consideration of selective pressures imparted by ecological dynamics, game theoretic issues arising from co-infections by non-identical strains, and biophysical constraints and trade-offs arising at the intracellular scale [50].

However, the first set of stochastic simulations of the phage GRN presented in this manuscript fail to predict the systematic decrease in the fraction of lysogeny given a fixed value of 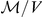 and increasing values of 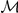 [29] (see Fig. 4). We revisited the original single-cell data and demonstrated the existence of an alternative scaling collapse owing to a proposed partial gene dosage compensation mechanism. When we incorporate partial gene dosage compensation within our stochastic model, we are able to recover the alternative scaling collapse consistent with the empirical measurements of Zeng *et. al.* [29] (see Fig. 5(D) and 6(B)). What might cause partial dosage compensation to occur in multiple infected cells? In stochastic simulations here, dosage compensation is modeled explicitly at the transcriptional level, whereas in reality multiple factors can contribute to it, and may occur at both transcriptional and post-transcriptional levels. The degree of compensation might change depending on copy numbers of genes and chromosomes as well as other intracellular factors. Copy number variation (CNV) is common in biological organisms [51], [52], and previous studies suggested that gene expression can depend sensitively on CNV when uncompensated [53]. Indeed, one hypothesis is that gene regulatory networks have been selected for their lack of dosage sensitivity to avoid problems in gene expression that may arise when CNV occurs naturally [54]. Previous studies showed that phage 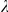 represses overall activity of RNA and protein synthesis within infected hosts depending on the number of coinfections [37], [38]. Viruses are known to control host cell cycle in eukaryotic cells [55], but how viruses affect the overall host transcriptional and translational activity in bacterial hosts is an open question. We believe that elucidating intracellular mechanisms of gene dosage compensation would be an important step toward understanding CNV and its resulting change in gene expression, at both the transient and steady state. In doing so, we also hope to provide a cautionary note: deducing explicit mechanisms from data collapses can be difficult, particularly when multiple data collapse schemes are consistent with observations.

In summary, this study proposed a novel intracellular decision-making mechanism to explain the variability in cell fate determination in multiply infected hosts. However, there can be other sources of variability underlying the lysis-lysogeny decision switch. First, the viral concentration, 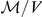, in naturally infected hosts may be dynamic. Multiple phages infect a host sequentially, and a host can keep growing while being infected. Subsequent infections increase 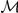 over time, and infected cells may spend a substantial fraction of the time prior to cell fate determination with a value of 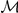 which is smaller than the final 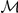. Next, host cell growth decreases 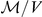 whereas viral genome replication increases 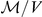 during the infection cycle. Clearly the dynamic nature of viral genome concentration needs to be addressed even if experimental protocols have been designed to synchronize infections. Second, despite our incorporation of stochasticity in the model, we assume the bacterial cytoplasm is well-mixed. Previous studies demonstrated that bacterial DNA, RNA and proteins have spatial patterns [56]–[58]. Bacteriophages are known to target cellular poles of hosts preferentially [59] which suggests phage genomes might be localized within bacterial cytoplasm. Hence, cell fate decision may be determined by local concentrations of regulatory proteins and quasi-independent cell fate determination by each virus. Finally, we assumed decision making as strict first passage processes arising from the consideration of thresholds as absorbing states within a GRN dynamics. It is possible that decision making involves soft thresholds over which cells make decisions with some probability. There are studies which show duration of signals is critical to cellular decisions [60], [61], and there might be some minimum time interval during which the system is above a threshold to make a decision [62]. Even if experimental protocols can minimize the impact of one of these mechanisms, the evolution of the phage 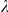 GRN would surely be impacted by all of them. Progress in identifying the importance of each of these issues at the molecular and evolutionary scales is relevant not only to the study of transient fate determination in phage 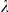, but to the study of cellular decision making in general.

## Models

### Gene regulation in phage 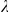


The fate of *E. coli* cells infected by phage 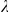 are decided soon after infection by a set of so-called early viral genes [4]. Among them we consider four genes, *cI, cro, cII* and *Q*, and one antisense mRNA (*aQ*) (see Fig. 1 (A)). The expression of these genes are controlled by four promoters, 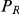, 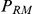, 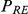 and 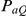. 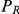 and 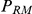 share three operator sites which are targeted by CI and CRO. The natural form of CI is a dimer, and CI dimers act as self activators and repressors for other genes by binding to 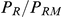. CII tetramers can bind to 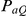 to transcribe 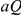 mRNA and 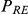 to produce CI [63]. Dimers of CRO bind to 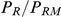 to inhibit all the genes in the system (Fig. 1 (B)).

Immediately after phage infections there are no viral gene products. At this initial stage 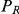 is active which leads to an increase of CRO, CII and Q levels. If Q becomes sufficiently abundant, it will turn on genes which make progeny phages, and the infected host will be lysed. However, as CII concentration increases CII tetramers can activate CI transcription from 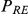, and CI expression level become further enhanced by the positive feedback loop of CI at 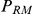. CII also represses Q by transcribing *aQ* which facilitates *Q* mRNA degradation, and sufficiently high CI level leads to lysogeny [4]. Hence, lysis or lysogeny is determined based on which of either CI and Q reaches the threshold concentration first. When CI reaches its threshold, CI dimers begin to form tetramers and octamers which lead to DNA looping [64]. DNA looping is very stable while maintaining lysogeny and repressing genes which trigger lysis [34]. When Q reaches its threshold, a group of late genes responsible for making progeny phages will be turned on, and the host will eventually be lysed. Since translation occurs with a single protein at a time, simultaneous crossings of lytic and lysogenic thresholds are forbidden, and the decisions are mutually exclusive. In reality, decisions would not be triggered by infinitesimally short bursts over decision thresholds, but for simplicity we assume a decision is made when either CI or Q concentration reaches its threshold for the first time. The use of step functions instead of Hill function type responses has been used extensively in the study of quantitative gene regulatory networks [16]. Note that when phages multiply infect cells in natural settings, they do not do so simultaneously, and so 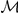 increases sequentially in time. However, for simplicity we only consider simultaneous coinfections, for which 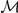 becomes a parameter in determining cell fate rather than a dynamic variable. This choice of modeling simultaneous infections is also motivated by the the experimental protocol of Zeng *et. al.* [29] in which rapid temperature changes were used to synchronize phage infection of DNA into host genomes.

### Quantitative model of phage 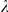 decision switch

Here we express the interactions among *cI, cro, cII* and *Q* as well as *aQ* mRNA described in the previous section as a set of ordinary differential equations. If we apply quasi-steady-state approximation for dimers and tetramers, the system can be described as



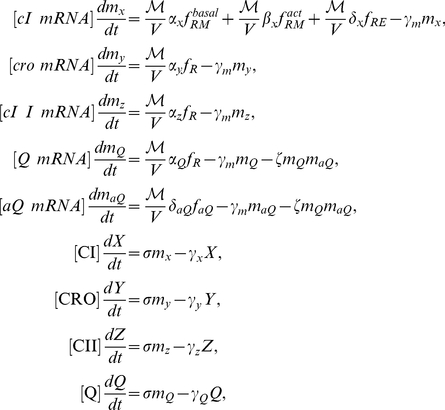
(3)


where 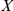, 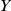, 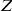 and 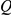 represent the total concentration of CI, CRO, CII and Q, respectively. 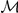 represents the number of coinfecting phages while 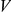 is the cell volume. 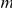 represents the mRNA concentration, and 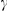 denotes the degradation rate where each subscript represent the species of associated gene/protein. *Q* and *aQ* mRNA become degraded by binding to each other and the adsorption rate is denoted as 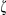. 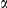, 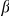 and 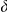 represent the basal, CI-mediated and CII-mediated transcription rates with subscripts indicating the species of mRNA. Note that 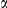, 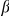 and 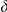 is inversely proportional to 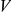 since the concentration change by a transcription event is proportional to 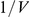. We assume that the concentrations of dimers and tetramers are at quasi-steady states such as 
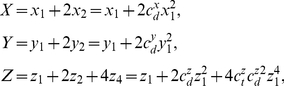
(4)


where the subscripts 1, 2 and 4 represent the concentration of monomers, dimers, and tetramers of each respective protein. 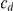 and 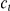 are the dimerization and tetramerization constants, respectively. 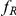, 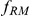, 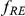 and 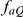 in Eq. (3) denote the probability of transcribable configurations for each promoters based on free energy change of possible states, and we follow the calculation of Shea and Ackers for 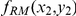 and 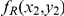 [36] and Arkin *et. al.* for 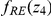 [25] (see Supplementary Text S1). 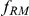 has two modes of transcription denoted as basal and activated depending on 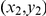. Response of 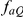 is a first order Hill function which is 
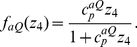
(5)


For stochastic simulations, we chose two parameter sets which lead to a transiently and asymptotically divergent lysis-lysogeny decision switch. Parameter values for the transiently divergent and asymptotically divergent cases are listed in Table 1. To calculate the fraction of lysogeny, we used at least 3,000 realizations of a stochastic model. Our simulations are based on Eq. (3) and are fully stochastic as implemented using the Gillespie algorithm [65] (see Supplementary Text S1 for details).

### Modeling gene dosage compensation

When gene dosage is compensated, the effective copy number, which is the fold change of transcription rate, is smaller than the actual copy number. Here we assume the effective copy number scales as 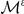 where 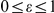. When 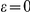, the system is completely compensated without any copy number dependence. On the contrary, when 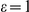, transcription rate is linearly proportional to the copy number. The experimental data (Fig. 5 (A)) supports that 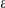 is between 0.4 and 0.6. For stochastic simulations, we replace all the terms of 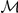 in Eq. (3) with 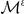, and set 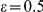.

## Supporting Information

Text S1 Additional details on methods.
(PDF)
