# Supplementary material for: To Lyse or Not to Lyse: Transient-Mediated Stochastic Fate Determination in Cells Infected by Bacteriophages
Source: PLoS Comput Biol. 2011 Mar 10;7(3):e1002006. doi: 10.1371/journal.pcbi.1002006 (PMC3053317; doi:10.1371/journal.pcbi.1002006)
Supplement: Text S1 — Additional details on methods. (PDF) [file pcbi.1002006.s001.pdf]

Text S1: Additional details on methods

Probability of an active promoter

We assume that promoters are in thermodynamics equilibrium with transcription factors, and given concentrations of transcription factors the probability of a specific configuration is constant. Table S1 and Table S2 list all the possible configurations of promoters. The probability of the i-th configuration is given as

$$f_i = \frac{exp(-\Delta G_i/RT)[CI_2]^{k_i}[RNAP]^{j_i}[CRO_2]^{l_i}}{\sum_i exp(-\Delta G_i/RT)[CI_2]^{k_i}[RNAP]^{j_i}[CRO_2]^{l_i}} \; for \; P_R/P_{RM} \tag{1}$$

$$g_i = \frac{exp(-\Delta G_i/RT)[CII_4]^{m_i}[RNAP]^{j_i}}{\sum_i exp(-\Delta G_i/RT)[CII_4]^{m_i}[RNAP]^{j_i}} \; for \; P_{RE} \,, \tag{2}$$

where G_i is free energy. f_i and g_i is the probability of i-th configuration for P_R/P_{RM} and P_{RE}, respectively. j_i, k_i, l_i and m_i represent the numbers of RNAP, CI, CRO and CII bound to the promoter. Note that a single RNAP occupies both O_{R1} and O_{R2} of P_R/P_{RM}. P_{RM} transcribes basally when $RNAP$ is bound at O_{R3}. When RNAP and CI binds to O_{R3} and O_{R2}, respectively, transcription by P_{RM} occurs at enhanced rate (activated). P_R is active only when RNAP is bound at O_{R2}/O_{R1} and O_{R3} is not occupied with CI. P_{RE} is only active when RNAP and CII binds to O_2 and O_1, respectively, hence $r4$ is the only configuration for possible CI transcription. Thus, the probability of transcribable configurations at each promoter, f_{RM}^{basal}, f_{RM}^{act}, f_R and f_{RE}, can be represented as

$$\begin{aligned} f_{RM}^{basal} &= f_{10} + f_{13} + f_{15} + f_{23} + f_{24} + f_{26} + f_{39} \,, \\ f_{RM}^{act} &= f_9 + f_{12} + f_{40} \,, \\ f_R &= f_{14} + f_{15} + f_{25} \,, \\ f_{RE} &= g_4 \,. \end{aligned} \tag{3}$$

We assume the concentration of RNAP within a cell is constant at 30 nM. The probabilities of activation for P_R, P_{RM} and P_{RE} as functions of total concentrations of CI and Q are shown in Fig. S1.

Stochastic simulation of phage λ switch

Eq. (3) represents the quantitative model of phage λ decision switch. Such a system of ordinary differential equations based on first order reaction kinetics can be turned into a stochastic model by using the Monte Carlo algorithm described by Gillespie [3]. All the reactions are listed in Table S3 (see Models for definitions of parameters). Note that f_{RM}^{basal}, f_{RM}^{act} and f_R are functions of CI and CRO dimer concentrations while f_{RE} and f_{aQ} are functions of CII tetramer concentration. To demonstrate the response to viral concentration ($\mathcal{M}/V$), we vary V from 0.5 to $2\mu m^3$. V is an explicit parameter within reaction rates, and it also indirectly affects the system dynamics since the concentration change by a transcription, translation and degradation event is linearly proportional to $1/V$.

Effect of thresholds on decision making

Thresholds are key parameters within first passage process models. In this section, we show how decision thresholds affect the functional and temporal characteristics of cell fate decisions. Recall that CI and Q threshold concentrations are associated with binding affinity of transcription factors and probability of forming a DNA loop [4], and may differ between viral strains. For the first set of analysis in Results the

Table S1. Configurations of P_R/P_{RM} and their total free energies [1].

State	O_{R3}	O_{R2}	O_{R1}	Free Energy (kcal)
$s1$	$\emptyset$	$\emptyset$	$\emptyset$	0
$s2$	$\emptyset$	$\emptyset$	CI	-11.7
$s3$	$\emptyset$	CI	$\emptyset$	-10.1
$s4$	CI	$\emptyset$	$\emptyset$	-10.1
$s5$	$\emptyset$	CI	CI	-23.7
$s6$	CI	$\emptyset$	CI	-21.8
$s7$	CI	CI	$\emptyset$	-22.2
$s8$	CI	CI	CI	-33.8
$s9$	RNAP	CI	$\emptyset$	-21.6
$s10$	RNAP	$\emptyset$	CI	-23.2
$s11$	CI	RNAP	RNAP	-22.6
$s12$	RNAP	CI	CI	-35.2
$s13$	RNAP	$\emptyset$	$\emptyset$	-11.5
$s14$	$\emptyset$	RNAP	RNAP	-12.5
$s15$	RNAP	RNAP	RNAP	-24.0
$s16$	$\emptyset$	$\emptyset$	CRO	-10.8
$s17$	$\emptyset$	CRO	$\emptyset$	-10.8
$s18$	CRO	$\emptyset$	$\emptyset$	-12.1
$s19$	$\emptyset$	CRO	CRO	-21.6
$s20$	CRO	$\emptyset$	CRO	-22.9
$s21$	CRO	CRO	$\emptyset$	-22.9
$s22$	CRO	CRO	CRO	-33.7
$s23$	RNAP	CRO	$\emptyset$	-22.3
$s24$	RNAP	$\emptyset$	CRO	-22.3
$s25$	CRO	RNAP	RNAP	-24.6
$s26$	RNAP	CRO	CRO	-33.1
$s27$	$\emptyset$	CRO	CI	-22.5
$s28$	$\emptyset$	CI	CRO	-20.9
$s29$	CI	$\emptyset$	CRO	-20.9
$s30$	CRO	$\emptyset$	CI	-23.8
$s31$	CI	CRO	$\emptyset$	-20.9
$s32$	CRO	CI	$\emptyset$	-22.2
$s33$	CRO	CI	CI	-35.8
$s34$	CI	CRO	CI	-32.6
$s35$	CI	CI	CRO	-33.0
$s36$	CI	CRO	CRO	-31.7
$s37$	CRO	CI	CRO	-33.0
$s38$	CRO	CRO	CI	-34.6
$s39$	RNAP	CRO	CI	-34.0
$s40$	RNAP	CI	CRO	-32.4

thresholds were set at 100 nM for both CI and Q (later we modified this when considering the partial gene dosage compensation mechanism). The fraction of lysogeny as a function of $\mathcal{M}$ can be changed by tuning thresholds (see Fig. S2(A) for $\mathcal{M} = 1$). We find that the fraction of lysogeny increases for all $\mathcal{M}$ by lowering the CI threshold and increasing the Q threshold for a given $\mathcal{M}/V$. On the contrary, a lower

Table S2. Configurations of P_{RE} and free energies [2].

State	O_1	O_2	Free Energy (kcal)
r1	$\emptyset$	$\emptyset$	0
r2	$\emptyset$	RNAP	-9.9
r3	CII	$\emptyset$	-9.7
r4	CII	RNAP	-21.5

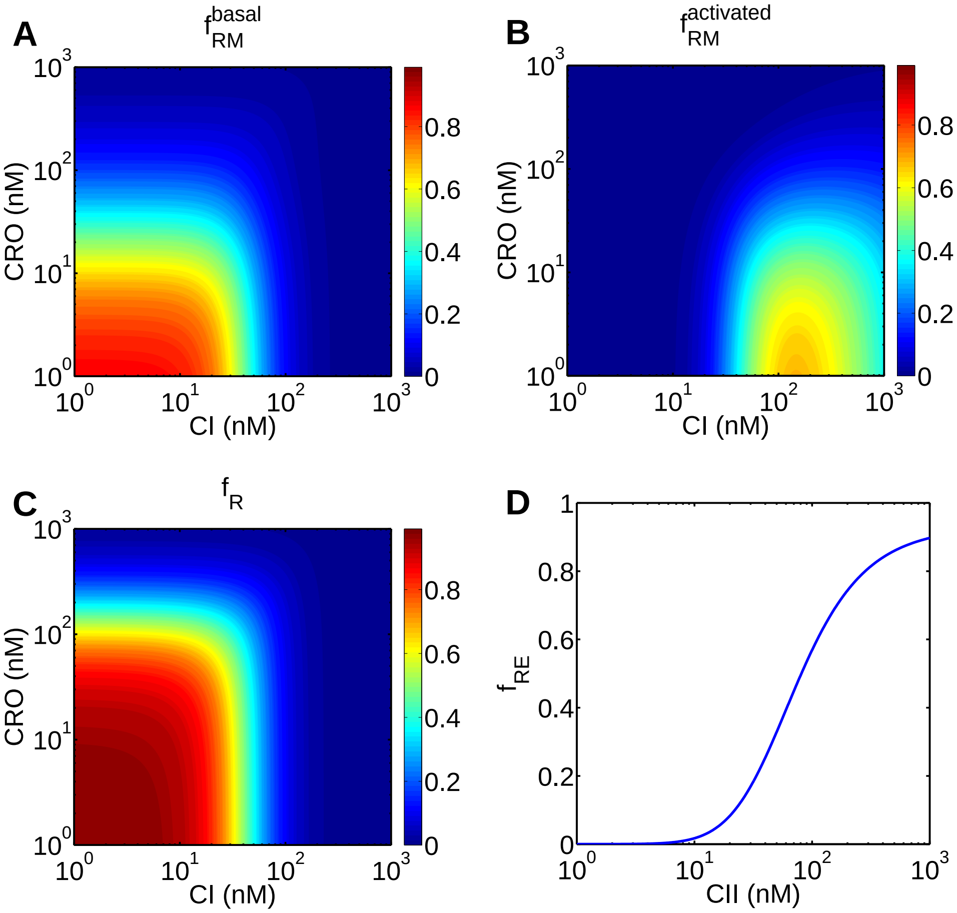

Figure S1. Probability of transcription initiation by (A) basal and (B) activated P_{RM}, (C) P_R and (D) P_{RE} as functions of total transcription factor concentrations. Note that f_{RM} and f_R is a function of CI and CRO dimer concentrations whereas f_{RE} is a function of CII tetramer concentration. By using quasi-steady-state approximation among monomers, dimers and tetramers, a fixed value of total concentration denotes a unique value of dimer/tetramer concentration.

Q threshold or higher CI threshold leads to a smaller fraction of lysogeny. Note that the response to viral genome concentration is determined by the fractions of lysogeny across various $\mathcal{M}/V$, and thresholds change the fraction of lysogeny for all $\mathcal{M}/V$. Thus, even if decisions can be tuned to be almost lytic or lysogenic at a given $\mathcal{M}/V$, there might be a limit of how sensitive the fraction of lysogeny can be

Type of reactions	Interacting species and total reaction rate		
Basal cI transcription by P_{RM}	cI	$\xrightarrow{\mathcal{M}\alpha_x f_{RM}^{basal}}$	$cI + mRNA_{cI}$
Activated cI transcription by P_{RM}	cI	$\xrightarrow{\mathcal{M}\beta_x f_{RM}^{act}}$	$cI + mRNA_{cI}$
cI transcription by P_{RE}	cI	$\xrightarrow{\mathcal{M}\delta_x f_{RE}}$	$cI + mRNA_{cI}$
cro transcription	cro	$\xrightarrow{\mathcal{M}\alpha_y f_R}$	$cro + mRNA_{cro}$
cII transcription	cII	$\xrightarrow{\mathcal{M}\alpha_z f_R}$	$Q + mRNA_{cII}$
Q transcription	Q	$\xrightarrow{\mathcal{M}\alpha_Q f_R}$	$Q + mRNA_Q$
aQ transcription	aQ	$\xrightarrow{\mathcal{M}\delta_{aQ} f_{aQ}}$	$aQ + mRNA_{aQ}$
$mRNA_{cI}$ degradation	$mRNA_{cI}$	$\xrightarrow{\gamma_m m_x V}$	$\emptyset$
$mRNA_{cro}$ degradation	$mRNA_{cro}$	$\xrightarrow{\gamma_m m_y V}$	$\emptyset$
$mRNA_{cII}$ degradation	$mRNA_{cII}$	$\xrightarrow{\gamma_m m_z V}$	$\emptyset$
$mRNA_Q$ degradation	$mRNA_Q$	$\xrightarrow{\gamma_m m_Q V}$	$\emptyset$
$mRNA_{aQ}$ degradation	$mRNA_{aQ}$	$\xrightarrow{\gamma_m m_{aQ} V}$	$\emptyset$
$mRNA_Q$ degradation by $mRNA_{aQ}$	$mRNA_Q + mRNA_{aQ}$	$\xrightarrow{\zeta m_Q m_{aQ} V}$	$\emptyset$
CI degradation	CI	$\xrightarrow{\gamma_x X V}$	$\emptyset$
CRO degradation	CRO	$\xrightarrow{\gamma_y Y V}$	$\emptyset$
CII degradation	CII	$\xrightarrow{\gamma_z Z V}$	$\emptyset$
Q degradation	Q	$\xrightarrow{\gamma_Q Q V}$	$\emptyset$
CI translation	$mRNA_{cI}$	$\xrightarrow{\sigma m_x V}$	$mRNA_{cI}$+CI
CRO translation	$mRNA_{cro}$	$\xrightarrow{\sigma m_y V}$	$mRNA_{cro}$+CRO
CII translation	$mRNA_{cII}$	$\xrightarrow{\sigma m_z V}$	$mRNA_{cII}$+CII
Q translation	$mRNA_Q$	$\xrightarrow{\sigma m_Q V}$	$mRNA_Q$+Q

Table S3. Stochastic reactions of transcription, translation and degradation. X, Y, Z and Q represent total concentrations of CI, CRO, CII and Q, respectively where as m_x, m_y, m_z, m_Q and m_{aQ} are mRNA concentrations. V is the volume of the infected host cell. See Models for description of other parameters

to the change of the viral genome concentration. So far we mainly focused on the fraction of lysogeny, the functional aspect of alternative decision making, but threshold concentrations also determine the speed at which decisions are made. Given fixed kinetic parameters, it takes less time to produce a small amount of viral proteins, so lower thresholds lead to faster decision time (see Fig. S2(B)). Extreme values of thresholds lead to shorter decision times but also tend to produce non-heterogeneous decisions as a function of variation in extrinsic parameters. Hence, faster decisions may come at the expense of the ability of the viral GRN to bias cell fate determination as a function of the extrinsic parameter, $\mathcal{M}/V$.

References

[1] Shea MA, Ackers GK (1985) The O_R control system of bacteriophage lambda. A physical-chemical model for gene regulation. J Mol Biol 181: 211-230.

[2] Hoyt MA, Knight DM, Das A, Miller HI, Echols H (1982) Control of phage lambda development by stability and synthesis of *cii* protein: role of the viral *cii* and host *hfla*, *hima* and *himd* genes. Cell

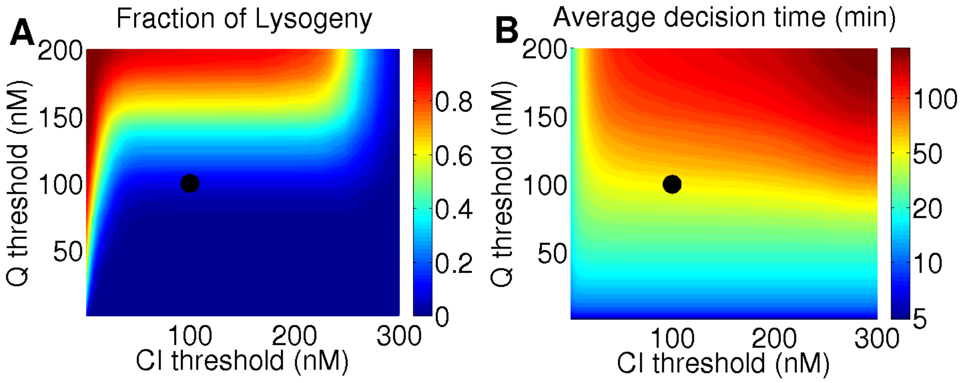

Figure S2. Effect of thresholds on decision making. (A) Functional (fraction of lysogeny) and (B) temporal (mean decision time) effect of thresholds at $\mathcal{M} = 1$ when transiently divergent. Black circle shows CI and Q threshold for simulation in the previous section.

31: 565-573.

[3] Gillespie DT (1977) Exact stochastic simulation of coupled chemical reactions. J Phys Chem 82: 2340-2361.

[4] Vilar JMG, Saiz L (2005) DNA looping in gene regulation: from the assembly of macromolecular complexes to the control of transcriptional noise. Curr Opin Genes Dev 15: 136-144.
